# Using Answer Set Programming to Integrate RNA Expression with Signalling Pathway Information to Infer How Mutations Affect Ageing

**DOI:** 10.1371/journal.pone.0050881

**Published:** 2012-12-10

**Authors:** Irene Papatheodorou, Matthias Ziehm, Daniela Wieser, Nazif Alic, Linda Partridge, Janet M. Thornton

**Affiliations:** 1 European Molecular Biology Laboratory–European Bioinformatics Institute, Wellcome Trust Genome Campus, Hinxton, Cambridge, United Kingdom; 2 Institute of Healthy Ageing, and Department of Genetics Evolution and Environment, University College London, London, United Kingdom; 3 Max Planck Institute for Biology of Ageing, Cologne, Germany; Albert Einstein College of Medicine, United States of America

## Abstract

A challenge of systems biology is to integrate incomplete knowledge on pathways with existing experimental data sets and relate these to measured phenotypes. Research on ageing often generates such incomplete data, creating difficulties in integrating RNA expression with information about biological processes and the phenotypes of ageing, including longevity. Here, we develop a logic-based method that employs Answer Set Programming, and use it to infer signalling effects of genetic perturbations, based on a model of the insulin signalling pathway. We apply our method to RNA expression data from *Drosophila* mutants in the insulin pathway that alter lifespan, in a *foxo* dependent fashion. We use this information to deduce how the pathway influences lifespan in the mutant animals. We also develop a method for inferring the largest common sub-paths within each of our signalling predictions. Our comparisons reveal consistent homeostatic mechanisms across both long- and short-lived mutants. The transcriptional changes observed in each mutation usually provide negative feedback to signalling predicted for that mutation. We also identify an S6K-mediated feedback in two long-lived mutants that suggests a crosstalk between these pathways in mutants of the insulin pathway, *in vivo*. By formulating the problem as a logic-based theory in a qualitative fashion, we are able to use the efficient search facilities of Answer Set Programming, allowing us to explore larger pathways, combine molecular changes with pathways and phenotype and infer effects on signalling in *in vivo*, whole-organism, mutants, where direct signalling stimulation assays are difficult to perform. Our methods are available in the web-service NetEffects: http://www.ebi.ac.uk/thornton-srv/software/NetEffects.

## Introduction

A major challenge in systems biology is the integration of different types of knowledge about biological processes in order to gain insight into the functioning of the organism as a whole system. Experimental data can include detailed molecular quantification experiments and records of phenotypic traits. Much of the time, knowledge such as pathway information is incomplete and is described at varying levels of detail in published records. Experimental data are usually specific to the question they were designed to address, limiting their power to shed light on more general properties of the system or to address different questions. In many cases the choice of the biological system, limits the type of experiments that can be performed, therefore relying on computational methods to make additional inferences. As we accumulate more knowledge about biological processes, it is important to review past experiments in the light of new knowledge, in order to extract further understanding of the system. An additional challenge is to bridge the gap between knowledge of biological pathways, genetic perturbations with their effects on gene expression and the resulting phenotypes, in order to gain a more thorough understanding of biological mechanisms. In this study, we developed a method for integrating pathway with gene expression and phenotypic data sets, making inferences from them and checking their consistency. These include information on changes in gene expression resulting from different gene mutants, biological signalling pathway information at the protein level in the form of activation and inhibition between proteins, and results of phenotypic analyses of the mutant animals.

Ageing is the process of decline in organismal fitness over time. This process differs significantly across species, as well as between individuals of the same species, leading to a variety of different age-related phenotypes and lifespans. The molecular basis of lifespan has been under intense investigation in recent years, using laboratory model organisms such as yeast, the nematode worm *Caenorhabditis elegans*, the fruit fly *Drosophila melanogaster* and the mouse *Mus musculus*. The insulin/insulin-like growth factor signalling (IIS) pathway and the target of rapamycin (TOR) pathway have been found to be major determinants of lifespan in these organisms. Importantly, as well as extending lifespan through the forkhead transcription factor *foxo*, reduction of IIS activity increases health during ageing. Initial experimental work showed that worm mutants for *daf-2*, the single worm IIS receptor, live twice as long as controls [1], [2]. Similar results from various components of the IIS pathway have been observed in flies [3], [4] and mice [5]–[8]. These findings have emerged from genetic manipulations of the ageing process, by targeting specific components of the IIS signalling network and measuring the responses of lifespan and, in some cases, genome wide patterns of gene expression. For a review of experiment types in ageing and computational methods available for their analysis, see [9]. Given the complexity and incomplete knowledge of the signalling pathways involved, it is often difficult to examine the transcriptional changes in the context of the signalling pathway architecture.

Protein abundance is predominantly controlled at the level of translation [10]. A recent study by Schwanhäusser *et al* [11] found that about 40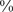 of variance in protein abundance is explained by mRNA levels in mammalian cells. This study showed that transcription factors and signalling molecules, in particular, have short mRNA and protein half-lives possibly because these are information carrying molecules, whose levels must be rapidly adjusted in response to environmental changes. Moreover, Nagaraj *et al* [12] report a good correlation of protein abundance with transcript abundance in cancer cell lines. It is therefore possible to make some, qualitative inferences about signalling pathways from mRNA levels in gene expression studies. In addition, genetic manipulations of the IIS pathway cause FOXO-mediated transcriptional responses in downstream pathways, as well as transcriptional feedback on upstream components of the IIS pathway [13], [14]. Previous studies (e.g. [15], [16]) report feedback at the transcriptional level, but this has not been examined across different mutants, longevity phenotypes and within the framework of the IIS and its neighbouring signalling pathways.

Here we develop a qualitative method that enables us to infer the impact of differential gene expression on signalling pathways and attempts to distinguish the effects of the primary experimental intervention from those caused by feedback. We achieve this by developing a comprehensive model of the pathways involved and a rule-based logical theory that integrates results from high-throughput gene expression assays with previous knowledge of the pathway. In this study we have used such data sets from *in vivo* experiments on ageing of the fruit fly *Drosophila melanogaster*, where direct experimental measurements of signal transduction by means of ligand stimulations are difficult to perform. Previous work on the inference of signalling pathway components from gene expression data sets has focused on inferring modulators of specific transcription factors by correlating mRNA expression profiles across large sets of microarray experiments [17], [18]. These methods rely on the availability of large sets of expression data and do not make use of any prior knowledge of the signalling pathway that modulates the transcription factor. In contrast, our approach combines previous knowledge of the pathway architecture and limited data on gene expression with logic, to obtain additional insights.

The modulation of small scale signalling pathways has previously been modelled by Ordinary Differential Equations (ODEs) that can represent chemical reactions. Networks of these equations can model biochemical processes in detail, provided there are appropriate experimental data sets to estimate the parameters of the model. This approach requires proteomic and phosphorylation experiments assessing the abundance of the different forms of the molecules so that the reaction parameters can be quantified. Although these models are very useful in describing specific reactions and small pathways [19], it is not very often that such experimental data sets are available for larger pathways with many nodes. Such ODE modelling is computationally intensive, so that simulations using networks larger than a few nodes are limited.

Methods using boolean logic and fuzzy logic have been successfully applied to simulate signalling pathways and study their activity under different conditions, such as targeted gene mutations or ligand stimulations. Calzone *et al* [20] use the software GinSim, [21], to model the regulation of cell-fate decision and identify conditions under which a cell decides to proceed with apoptosis, survival or non-apoptotic cell death using a network of 14 nodes, reduced from a literature-derived network of 28 nodes. These methods are based on the development of transition graphs, synchronous or asynchronous, although asynchronous transition graphs resemble more closely the signalling pathway mode of action. Although powerful for solving highly connected small networks with loops, the time required for analysis of these graphs is exponential, leading to computational difficulties when dealing with pathways containing larger numbers of nodes. Saez-Rodriguez *et al* [22] developed CellNetOpt to successfully optimise pathway models against high-throughput biochemical measurements of phosphorylation states. For a more comprehensive review on logic-based models, see [23].

Our approach uses Answer Set Programming (ASP), a methodology for declarative programming that supports logic-based inference, [24]. ASP provides a flexible and expressive framework for describing theories, and benefits from the development of efficient solvers, such as the Potsdam answer set solving collection [25]. ASP has been used to model the sulfur starvation-response pathway of *Arabidopsis thaliana* using action languages on ASP [26] and model cell cycle networks in yeast [27]. Later, Fayruzov *et al*, [28], adapted their ASP framework to behave as a boolean network. They conclude that their approach is more expressive than boolean networks, due to the flexibility of representing implicit assumptions and background knowledge, as well as the scalability for expressing special cases with the ASP framework. Recently, it was applied to the development of BioASP [29], a python library that provides tools for checking the consistency between experimental data and pathways and suggest new pathway links that could improve the fit between experiments and pathways. BioASP expresses and extends, with use of ASP, the Sign Consistency Model (SCM) that is employed by the cytoscape plug-in BioQuali [30]. Finally, Videla *et al*, [31], have recently compared the training of logic models of signalling networks using optimisation heuristics with a new implementation based of Answer Set Programming. Their assays showed significant improvements on computation time by the use of ASP. In addition, their ASP implementation was able to identify all possible solutions as opposed to the stochastic optimisation method.

In this study we constructed a model of the IIS signalling pathway including its interactions with the TOR pathway and developed a novel method for inferring the modulation of the pathways from large-scale gene expression data sets using data from different mutants affecting longevity. Our resulting rule-based theory enables the inference of effects in a large and complex pathway, implicitly checking experimental consistency, by using a single expression data set at a time. It is also possible to make a custom query of the pathway components the user wishes to perturb and predict the effects on signalling and the phenotype. By using qualitative data sets and an ASP solver, we avoid the computational limitations faced by other methods, and we are hence able to work with larger complex pathways that include feedback loops and make the methods available as a web-service. By using prior knowledge of the pathway connections, we overcome the requirement for a large number of experimental data sets for our inferences. Our analyses focus on experimental data sets from *Drosophila melanogaster* that manipulated the genes *chico*, *Lnk*, *foxo* and *InR* to produce a significant difference in lifespan between mutant and wild type strains. We analyse one experiment at a time and we also provide new methods for the comparisons of our inferences across experiments. The methods are implemented in a web-service, NetEffects, http://www.ebi.ac.uk/thornton-srv/software/NetEffects, allowing users to analyse their expression and longevity data using this approach, or to predict probable effects on longevity for mutations to any components of the pathway.

## Methods

### A model of the *Drosophila* insulin pathway and its interaction with the TOR pathway

**Figure 1:**
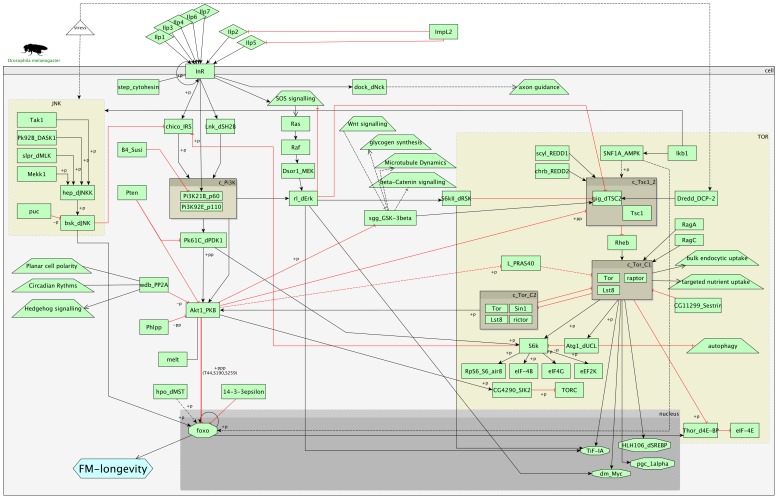
The insulin pathway in *Drosophila*. Insulin signalling pathway and its interconnections with the TOR pathway. The insulin pathway appears on the left-hand side towards the centre of the image, while the TOR pathway appears on the right-hand side. There are many connections facilitating cross-talk between the two pathways. Black arrows represent activating connections, while red-T-lines represent inhibitions. Green rectangular boxes represent proteins, while trapezoid boxes represent processes or pathways. We use the Flybase gene symbols as names but also include other commonly used names, in cases where these are appear more frequently in the literature. Protein-complexes are represented by boxes whose name always begins with “c_”. Transcription factors are shown by octagons, but only some examples are shown. The FM-longevity (FOXO-mediated longevity) phenotype is represented by a hexagon.

The IIS signalling pathway and its interactions with the TOR pathway are shown in Figure 1. The IIS pathway is a neuroendocrine pathway, whose single receptor, INR, can be activated by seven different insulin-like peptides (ILPs) present in *Drosophila*. INR activation leads to the recruitment of the single *Drosophila* insulin receptor substrate CHICO and of the *Drosophila* homologue of human SH2B, called LNK. These proteins act in parallel and activate the phosphotidylinositol-3-kinase (PI3K) complex formed by proteins P60 and P110 [32]. The PI3K activates the phosphoinositide-dependent kinase-1 PDK1, leading to the activation of the protein kinase B, AKT1, which inhibits the activity of the forkhead transcription factor FOXO. This core signal transduction pathway is modulated by numerous other proteins, such as the cytohesin, STEP, [33], B4/SUSI [34], PHLPP [35], or the IGFBP7 homologue imaginal morphogenesis protein-late-2 (IMP-L2) [36].

The insulin pathway interacts with other signalling pathways, such as the JNK kinase, SOS signalling and Wnt pathways. It is also highly interconnected with the target-of-rapamycin (TOR) pathway, mainly mediated through CHICO, PI3K and AKT1 in the IIS and the TSC-complex, L (LOBE), TOR-C2 and S6K in the TOR pathway. The core TOR pathway is triggered by low intracellular AMP levels, which activate the AMP kinase SNF1A, which in turn activates the TSC-complex. The complex inhibits RHEB, which then no longer activates the TOR-C1 complex. The TOR-C1 complex is a major signalling complex integrating various signals and activating and inactivating numerous proteins amongst which are transcription factors such as the *Drosophila* MYC/DM, as well as signalling proteins, such as S6K.

The complex network of these interactions has been manually compiled from pathway diagrams of the KEGG database [37], [38] and an extensive literature review based on review articles [39], [40], as well as the primary literature, e.g. [41], [42]. In cases of doubt or incomplete information, interactions and the logical connections have been cross checked for experimental evidence in *Drosophila melanogaster*. While we have tried to cover the two pathways and their interactions as completely as possible, the diagram is undoubtedly incomplete because of the limits of biological knowledge.

The diagram has been created in GraphML using the program yEd (http://www.yworks.com). This is an XML based format, which allows a formal interpretation, while at the same time defining a graphical layout. It thus enables a graphical representation, which is immediately understandable to biological readers and at the same time allows computational use of the same file. The diagram shows activation and inhibition relationships of the protein components of the signalling network, thus representing the activity wiring. Metabolic, transcriptional or translational influences are not considered. For tractability, we do not include any kinetic data or location-specific information in the pathway. These compromises in complexity are essential to allow the examination of such a large and complex signalling network. However, our approach is far more detailed than pure protein-protein-interaction studies, which lack directionality and connection types (i.e. induces/inhibits).

### Finding signals from expression data sets

We applied our methods to microarray gene expression studies of three different genetic null mutants (*chico* ^1^ */+, Lnk* 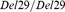 *, dfoxo* 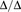) and one loss of protein function (*daGAL4* 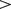 *UAS-dInR* 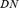) in components of the IIS pathway. Three of those conditions, *chico* (*chico* ^1^ */+*), *Lnk* (*Lnk* 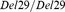) and *InR* (*daGAL4* 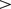 *UAS-dInR* 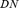), result in lifespan-extension of *Drosophila melanogaster*. The fourth condition, *foxo*, (*dfoxo* 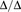) results in lifespan-reduction and is a homozygous deletion of the forkhead box O transcription factor, whose function is inhibited by the activation of insulin signalling. The *Lnk* experiment generated homozygous mutants null for the gene *Lnk*, whereas the *chico* experiment generated a heterozygous mutant of the gene *chico*. The *InR* experiment involved an over-expression of a dominant negative form of the gene *InR*. RNA expression microarray assays were performed on whole flies for all experiments, except for *Lnk*, where the assays where performed on fly heads only. These studies are publicly available in [15], [16], [43].

**Figure 2:**
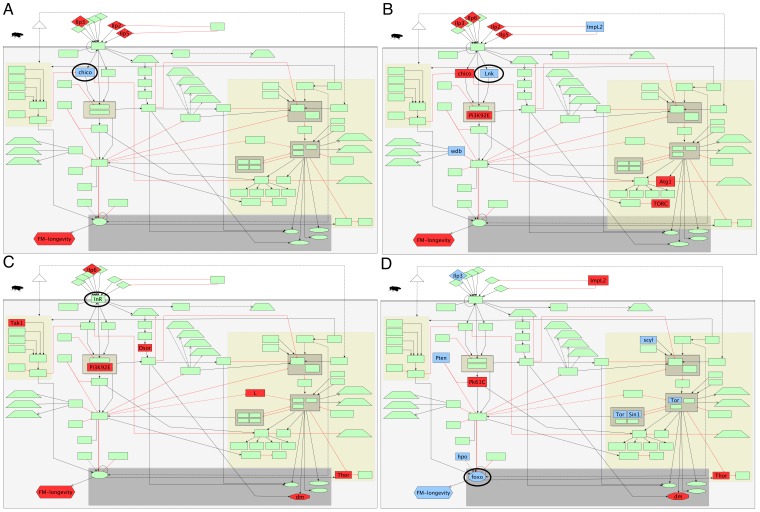
Differentially expressed components of the insulin pathway for each experiment. Down-regulated genes appear in blue, whereas up-regulated genes appear in red. Circled nodes represent the genetically manipulated components. Image (A) corresponds to the experiment that mutated *chico*, (B) to *Lnk*, (C) to *InR* and (D) to *foxo*.

Each microarray data set was analysed, to identify the set of differentially expressed genes. Raw data sets were summarised and normalised using RMA [44]–[46] and quantile normalisation as implemented in the LIMMA package. Differential expression between mutants and wild-type controls was assessed using linear models and the empirical Bayes moderated *t*-statistic implemented in LIMMA [47]. Highly differentially expressed genes were selected by applying a 0.005 cutoff on the adjusted *P*-value for each experiment. The analysed data sets are available in supplementary file S4. In Figure 2 we show which genes, in the IIS and TOR pathways, are differentially expressed in each experiment.

**Figure 3:**
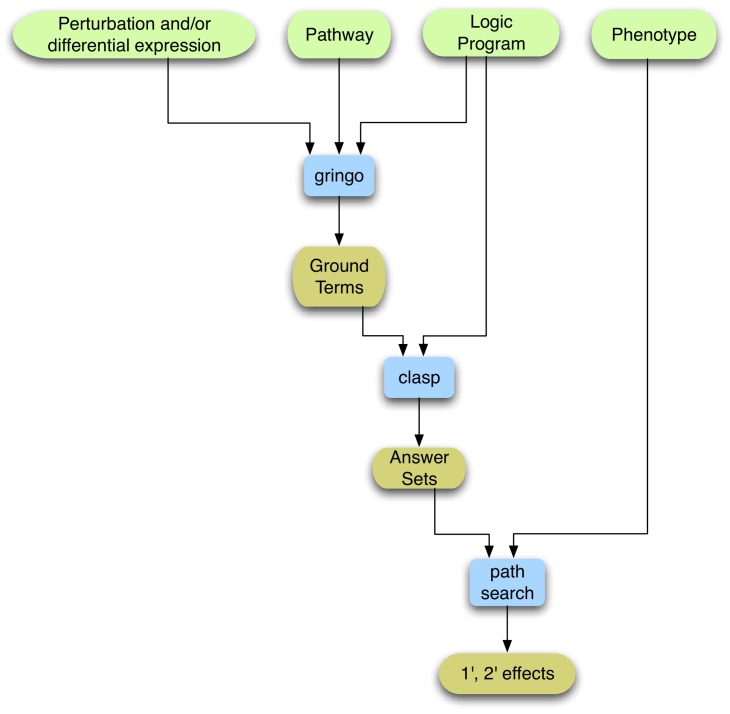
Flow chart of methods and data types. For the ‘grounding’ stage of ASP, with the tool ‘gringo’, we use our rule based representation (logic program), the pathway relations and the appropriately encoded gene expression data sets (facts). Gringo, then produces all possible ground facts, which are then handled by the solver, ‘clasp’, which uses the logic program with the integrity constraints to find consistent ‘answer sets’. Finally, using our own custom made program for path searches, we process the answer sets to identify affected paths in the experiment using each differentially expressed or mutant gene as a starting point and longevity as the end point. These paths are split into primary or secondary effects using the information on the longevity phenotype, acquired by survival assays, complementary to each experiment.

**Figure 4:**
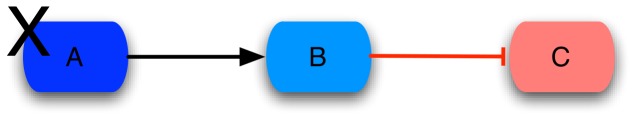
Example of applying the ASP methods on a short pathway. This example pathway consists of three components which are linked with two signalling interactions, where A induces B and B inhibits C. If A is knocked out in an experiment, as shown by the symbol ‘X’, then the expression of the transcript of A will decrease (dark blue). The effect of this decrease in expression, at the protein level of this pathway, is that B (light blue) will remain inactivated by A and C (light red) activated as a result of lack of inactivation by B.

Then we used the workflow summarised in Figure 3, in order to infer possible signalling paths within each experiment. We used as inputs the pathway and the microarray results, together with information on the experimental perturbation to build a knowledge base of facts for each experiment. Using the general logic program that links those facts with potential outcomes for the signalling of the proteins involved, we inferred how the perturbation and subsequent differential expression could influence the activation of the different pathway components at the signalling level. Figure 4 illustrates this process by use of a small example. This step uses ASP and consists of the two steps of ‘grounding’, augmenting the facts by use of the logic program, as described in Supplementary Files S2 and S5, that in essence assigns values to the variables according to the facts and rules, and ‘solving’, filtering the ground sets using the rules and integrity constraints to find consistent answer sets. We used the publicly available grounder ‘gringo’ and solver ‘clasp’, as described in the Supplementary File S5, to output sets of ‘activates’ and ‘inactivates’ relations. Each one of the experiments yielded one answer set.

The final step involved the inference of paths using each differentially expressed or perturbed pathway component as a starting point and longevity as an endpoint. For most differentially expressed or perturbed components, there is a large number of paths leading to the phenotype. This is due to the several connections between the IIS and the TOR pathways that lead to the generation of loops. We use the shortest paths in some of the analyses to show the direct effects of the perturbation or differential expression to the phenotype. We also use all the paths generated in order to derive feedback routes that could play a role in the signalling pathway modulation within each mutant (analyses shown in later sections). All paths are available in Supplementary File S1 and the shortest paths per experiment are shown in Table 1.

**Table 1:** Shortest paths of potentially active primary and secondary effects inferred per experiment.

chico	Primary Effects	chico 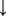 Pi3K 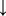 Akt1 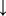 foxo 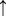 longevity 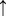
	Negative Feedback	Ilp2 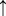 InR 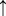 Lnk 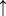 Pi3K 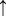 Akt1 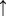 foxo 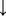 longevity 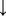
		Ilp3 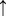 InR 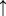 Lnk 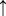 Pi3K 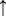 Akt1 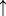 foxo 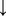 longevity 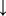
		Ilp5 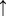 InR 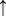 Lnk 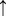 Pi3K 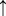 Akt1 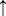 foxo 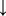 longevity 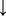
Lnk	Primary Effects	Lnk 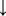 Pi3K 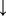 Akt1 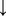 foxo 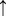 longevity 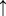
	Negative Feedback	Ilp2 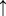 InR 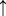 chico 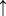 Pi3K 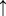 Akt1 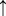 foxo 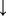 longevity 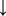
		Ilp3 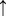 InR 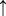 chico 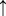 Pi3K 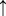 Akt1 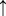 foxo 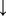 longevity 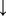
		Ilp5 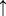 InR 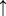 chico 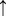 Pi3K 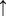 Akt1 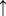 foxo 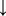 longevity 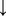
		Ilp6 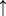 InR 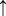 chico 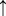 Pi3K 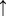 Akt1 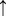 foxo 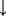 longevity 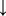
		ImpL2 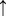 Ilp2 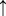 InR 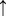 chico 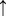 Pi3K 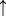 Akt1 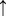 foxo 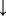 longevity 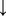
		ImpL2 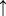 Ilp5 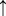 InR 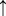 chico 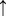 Pi3K 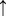 Akt1 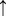 foxo 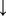 longevity 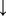
		wdb 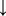 Akt1 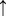 foxo 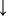 longevity 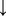
		chico 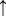 Pi3K 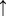 Akt1 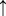 foxo 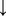 longevity 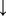
		Atg1 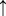 S6k 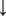 chico 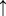 Pi3K 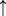 Akt1 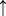 foxo 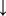 longevity 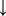
InR	Primary Effects	InR 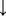 chico 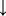 Pi3K 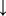 Akt1 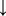 foxo 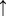 longevity 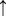
		InR 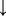 Lnk 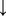 Pi3K 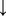 Akt1 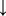 foxo 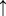 longevity 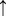
	Positive Feedback	Tak1 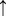 hep 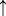 bsk 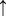 foxo 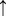 longevity 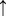
	Negative Feedback	L 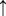 TOR-C1 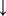 TOR-C2 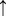 Akt1 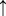 foxo 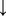 longevity 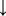
foxo	Primary Effects	foxo 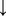 longevity 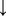
	Positive Feedback	Pk61C 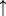 Akt1 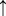 foxo 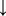 longevity 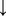
		hpo 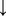 foxo 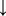 longevity 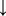
		Pten 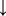 Akt1 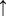 foxo 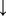 longevity 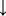

At this stage we used the phenotype recorded by the survival analyses that accompanied each experiment, in order to classify the inferred paths into ‘primary’ or ‘secondary’ effects. We call primary effects those paths that support the observed longevity phenotype and involve the primary experimental mutation. Secondary effects refer to paths that are triggered by differential expression of genes, other than the primary mutant. These are further classified into negative and positive feedback, where effects of negative feedback contradict the observed longevity phenotype, whereas positive feedback effects support the observed phenotype.

### Searching for longest common sub-paths

By comparing the different paths produced within and across the different experiments, we observed that many non-identical signals contained similar “segments” of paths that could potentially reveal important recurrent routes of activity in the signalling pathway. For example, each one of the long-lived mutants up-regulates different insulin-like peptides (ILPs) that in turn can lead to changes in longevity as mediated by FOXO (Table 2). When comparing whole paths between experiments, these similarities are missed, since the starting point (the ILP molecule) differs between paths, even though the rest of the path is the same. Moreover, by looking only at the shortest paths in an experiment, the information that can be derived from the rest of the paths is missed. We therefore developed a method to extract common sub-paths (routes) from all the paths per experiment and then compare these across experiments. These sub-paths highlight the routes that are accessible in a mutant, given the architecture of the pathway, the mutations and differentially expressed components.

**Table 2:** Signalling sub-path example.

Ilp2 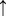 ***InR*** 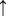 ***Lnk*** 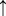 ***Pi3K*** 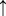 ***Akt1*** 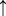 ***foxo*** 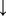 ***longevity*** 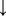
Ilp3 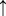 ***InR*** 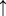 ***Lnk*** 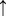 ***Pi3K*** 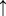 ***Akt1*** 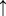 ***foxo*** 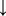 ***longevity*** 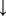
Ilp5 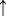 ***InR*** 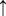 ***Lnk*** 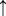 ***Pi3K*** 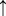 ***Akt1*** 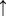 ***foxo*** 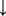 ***longevity*** 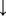

In this example there are three different signals that only differ by the type of ILPs that trigger INR and the rest of the IIS pathway via LNK. When comparing these, it is often useful to ignore the ILPs and use for our analyses the longest common string, which in this case is the signalling cascade downstream of each ILP.

In order to be able to identify the most common sub-paths within or across experiments, we developed a “longest common sub-path finder”. A sub-path is defined as a sequence of ‘activates’/‘inactivates’ relations that is part of a path that has a differentially expressed or perturbed component as a starting point and longevity as an end point. The longest common sub-path finder takes as input all the different paths produced by the analysis of the results from ASP and outputs a list of common sub-paths, together with their frequency in the data set. The algorithm uses an existing solution of the ‘longest common string’ program, implemented in the Perl package (String-LCSS-0.12, available from the Comprehensive Pearl Archive Network, www.cpan.org). Every path is compared to each other and the longest common sub-string is found for each comparison. The occurrences of each sub-string (sub-path) are counted and returned. The algorithm takes into account the sequence of nodes (pathway components), as well as their status (activated or inactivated). The longest common sub-path finder program is available for download from http://code.google.com/p/longest-common-sub-path.

## Results

### Identifying general trends between experiments

We observed that in all cases, the shortest paths starting from the mutated component support the observed phenotype, as expected, whilst most of the shortest paths starting from the differentially expressed components predict the opposite phenotype, suggesting negative feedback responses to the genetic perturbation, triggered at the transcriptional level.

We first compared the whole paths inferred by our methods across experiments. Only the experiments that mutated the *chico* and *Lnk* genes had some paths in common. This is not surprising, since these experiments mutate genes whose products function in a parallel fashion, by transmitting a signal from the receptor INR to the PI3K complex.

**Figure 5:**
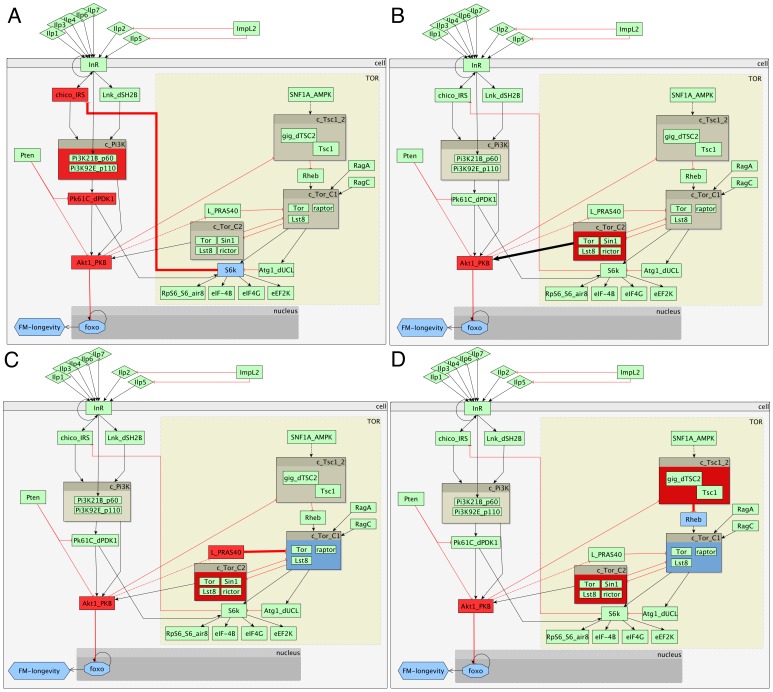
Links derived by sub-path comparisons and their suggested impact on longevity. (A) The link between the inactivated S6K and activated CHICO is found in several sub-paths in the *lnK* and the *InR* mutant and is suggested to act as a secondary effect, reducing lifespan by FOXO inhibition. (B) Another commonly found link in several sub-paths of *Lnk* and *InR* and some paths of *chico* is the activation of AKT1 by TOR-C2, suggesting a secondary effect and a crosstalk between TOR and IIS pathways. (C) The link of L inactivating TOR-C1 by inhibition is found in both *Lnk* and *InR*, as well some paths of *chico*. In the *InR* experiment *L* appears to be up-regulated, further supporting a possible secondary effect on longevity through TOR-C2 and AKT1. (D) Another route to AKT1 activation by TOR-C2 activation, also found in both *InR* and *Lnk* sub-paths, as well as some paths of *chico*, involved the inactivation of RHEB by the TSC1 and TSC2 complex.

Then, we produced the most common sub-paths for each experiment (available in supplementary file S3). These reveal the routes which most commonly occur in the response paths per experiment. Then, we compared the sub-paths across experiments, by asking three different questions. First, we looked for the most common sub-path in all long-lived mutants, which was the link from AKT1 to FOXO and longevity. The sub-paths of the *Lnk* and *InR* experiments were generally more similar than those in *chico*, although we did not observe any similarities between whole paths of *InR* and *Lnk*. The sub-path that exists in *Lnk* and *InR* but not in *chico*, corresponds to the secondary effect that reduces longevity through FOXO inhibititon via inactivation of S6K and subsequent activation of CHICO (Figure 5A). We looked into the common links between other sub-paths that we could detect in long-lived mutants, especially in *Lnk* and *InR*. These were various links that could lead to secondary effects, involving AKT1 activation by the TOR-C2 complex (Figure 5B), in some cases triggered by the inhibition of RHEB and subsequently TOR-C1 by the complex of TSC1 and TSC2 (Figure 5D), whereas in others by the inhibition of L on TOR-C1 (Figure 5C). All these effects are also detected within the paths inferred in *chico*, although they do not appear as longest common sub-paths. Gene *L* appears to be up-regulated in the *InR* mutant, where we infer a shortest path starting with L inhibition on TOR-C1, followed by TOR-C2 and AKT1 activation, leading to lifespan reduction by FOXO inactivation.

The current experiments and the connections on the pathway map are too few to identify striking differences across experiments. However, we do identify responses shared across all and one response shared by two out of three long-lived experiments. These routes could be of significance as they form part of the feedback mechanism that might be similar across experiments but triggered by different components. Specific examples are described in the next sections.

### Identification of opposite transcriptional feedback to insulin signalling in short- and long-lived mutants

We initially looked at the shortest paths from each mutant or differentially expressed component in the pathway to FOXO and longevity. Looking at the shortest active paths only, as shown in Table 1, we observe that primary effects in **long-lived** mutants suppress insulin signalling whilst secondary effects tend to stimulate the IIS pathway.

In contrast, in the *foxo* **short-lived** mutant we observe that the primary effect is reduction of lifespan, as *foxo* has been directly mutated. Therefore, no negative feedback can be inferred using the default settings of our method, since it would require FOXO being able to get activated. We do, however, observe differential expression on other components of the signalling pathways that would be consistent with suppressing insulin signalling by, for example, down-regulating *Ilp* molecules. We call these “impaired effects”, since one or more components of the path are mutated or differentially expressed in such a way that the signal is disrupted. In the case of the down-regulation of the insulin-like peptides that would normally suppress insulin signalling and activate FOXO, the signal is disrupted by the experimental knocking out of *foxo*. Moreover, in the *foxo* mutant we only infer positive feedback secondary effects that suppress FOXO.

In all three long-lived mutants some of the *Ilp* molecules are up-regulated, leading to FOXO inactivation, by retention in the cytoplasm and potentially a reverse effect to the observed lifespan-extension. For instance, *Ilp2*, *Ilp3* and *Ilp5* are up-regulated in the *chico* knock out, *Ilp2*, *Ilp3*, *Ilp5* and *Ilp6* appear up-regulated in the *Lnk* mutant and *Ilp6* is up in the *InR* mutant. In contrast, we observed *Ilp3* to be down-regulated in the short-lived *foxo* mutant, leading to an “impaired” path that aims to activate FOXO by suppressing insulin signalling.

Similarly, *ImpL2*, which inhibits ILP2 and ILP5 [36], appears to be down-regulated in the long lived *Lnk* mutant, while it is up-regulated in the short-lived *foxo* mutant. The aim, in this case too, appears to be the reduction of the inhibition of insulin signalling in the long-lived mutant, so that normal FOXO activity levels are restored, while in the short-lived mutant, the insulin signalling is reduced by inhibition of the ILPs. Hence, feedback in vivo involves extracellular components, such as one or more of the ILPs or IMPL2.

Looking at the signalling sub-paths we inferred for each experiment, we also observed that the TOR-C2 inhibition by the down-regulation of *Tor* and *Sin1* in the *foxo* mutant could lead to inactivation of AKT1 and consequent FOXO activation, provided *foxo* was not knocked out, and lifespan extension. This effect is not observed at all in the *Lnk* mutant, where we only observe the opposite, TOR-C2 and AKT1 activation. In the *chico* mutant we observe no sub-paths containing the link between TOR-C2 and AKT1 and in the *InR* mutant we observe many occurrences of activation of TOR-C2 and AKT1 and a few cases of inactivation of TOR-C2 and AKT1, in long sub-paths that contain loops through the IIS pathway (AKT1) and various players of the TOR pathway (L, TSC1, TSC2, TOR-C2 and TOR-C1).

Overall, these effects show a strong negative feedback that is consistent amongst long-lived mutants and opposite between short- and long-lived mutants. This type of negative feedback leads to homeostasis, in an attempt to reverse the effects of the primary mutations.

### An S6K-CHICO mediated feedback is identified in the long-lived fly mutants in vivo

Looking at the shortest paths derived from the *Lnk* mutant, we identified a novel hypothesis that involves a possible feedback (secondary effect) route from the up-regulation of *Atg1* to lifespan reduction via S6K inactivation and CHICO activation, uncovering a possible cross-talk between IIS and TOR signalling (Table 1). Although none of the genes whose proteins take part in this signal are differentially expressed in the *InR* mutant, we observed sub-paths containing the S6K signal (Figure 5), suggesting that it could play a role in this mutant too. This signal was absent from our third long-lived mutant, *chico*, since it requires that chico is activated, impossible in the *chico* knock out.

Partial support for this hypothesis comes from a study in [48], who demonstrated that when *Tsc1* and *Tsc2* are knocked out, there is a negative feedback to CHICO and ultimately AKT1 via S6K. In the case of the *Lnk* mutant we do not observe a down-regulation of *Tsc1* or *Tsc2*, but we observe an up-regulation of *Atg1*, which acts downstream of TSC1/TSC2. It would be interesting to investigate whether this negative feedback via S6K is indeed active in the *Lnk* mutant. Finally, it has been shown that the ribosomal S6 kinase 1 deletion causes lifespan extension in mice [8]. Although S6K1 inhibits the insulin pathway in mice [49], lifespan-extension most likely occurs by a different route rather than by interference with the insulin pathway and FOXO given that there are no known outgoing connections from the S6K or any downstream connections towards the IIS, except for the inhibition of CHICO by S6K. It would be interesting to compare the IIS and TOR pathways and equivalent experiments between the two species, in order to understand in depth the mechanisms under which S6K contributes to lifespan and where these intersect across species.

### Identifying contradictions and targets for further work

We observed that *Thor* and *myc*, both targets of the TOR pathway, but also targets of other pathways, appear to be up-regulated both in the short-lived *foxo* flies, as well as in one of the long-lived mutants (*InR*). A possible hypothesis is that they do not play a primary role in lifespan and there is another process regulating them that is currently not included in our pathway model. Another example of missing information is gene *L*, also known as *lobe*, which is involved in a cross-talk between AKT1 in the IIS pathway and the TOR pathway, TOR-C1 complex. In our examples, we observed *L* to be up-regulated in the *InR* mutant, where it is suggested to be involved in a secondary effect by providing feedback to AKT1 through the TOR complex. According to its functional description in Flybase (ID: FBgn0001332), it also appears to be implicated in the regulation of the Wnt signalling and of the JNK cascade, although there is no evidence of the underlying mechanism.

In the *InR* experiment we also observed up-regulation of *Tak1*, a component of the JNK pathway. This pathway is ubiquitous, in being involved in many different biological processes. It has been implicated in lifespan through FOXO activation [50]. The mechanism under which the JNK signals integrate with the IIS signals is unclear, but the JNK pathway is known to antagonise the IIS. In our example, TAK1 leads to a positive feedback effect by activating FOXO. Other examples of such positive feedback effects are *Pk61C* up-regulation and the *hpo* and *Pten* down-regulations in the *foxo* knock-out experiment. All three of them lead to an inactivation of FOXO according to our current pathway knowledge, indicating possible points for further research.

We could not identify any direct paths to FOXO and longevity from *p110*, the kinase subunit of the PI3K complex, but we noticed that it is over-expressed in two different lifespan-extending experiments. We believe that this highlights the need for further investigations on this kinase and its role in ageing via insulin pathway perturbations.

## Discussion

Our method successfully combines knowledge of the IIS and TOR pathways with gene expression data sets and longevity assays, to make inferences about the modulation of the signals in different mutants of the IIS pathway and explain the observed phenotypes. Such insight in the modulation of signal transduction cannot routinely be gained experimentally from such *in vivo* experiments, although it is important in order to be able to uncover the complexities of ageing. A strength of our approach is that it enables us to investigate data sets from many experiments in a fast, simple and rigorous way, uncover detailed paths per experiment, as well as consistent biological trends across experiments, such as the homeostatic behaviour observed across the different mutants. Such insights cannot be produced by standard functional analysis methods for gene expression data sets. Moreover, the application of a computationally efficient and scalable method such as ASP enables the development of web-services, making such complex analyses easier to perform and accessible to experimental scientists.

An assumption that our pathway model makes is that longevity is solely a result of FOXO activation via the insulin pathway, which is primarily a nutrient sensing pathway coordinating appropriate responses to nutrient availability. Although extensive, our pathway model ignores further complexities of the interactions with other pathways or other determinants of lifespan. By including components of the TOR pathway in our model, especially where this cross-talks with the IIS pathway, we were able to identify how TOR could provide feedback into the IIS pathway under the conditions of certain IIS perturbations. However, the TOR pathway has also been implicated in lifespan possibly through different routes, as demonstrated in other organisms [8]. Moreover, it is unknown how the gene targets of FOXO affect lifespan in the fly. Such information would enable a more complete analysis and a better interpretation of the results on the *foxo* mutant. Although the majority of our results are consistent with previous knowledge and we are still able to generate interesting hypotheses for further investigations, we can not assign roles or causes for the differential expression of genes that trigger positive feedback with respect to FOXO-mediated longevity.

By formulating our problem as a logic-based theory, we are able to use the efficient search facilities of Answer Set Programming and can therefore explore larger pathways. Secondly, we take advantage of the rich and expressive language of formal logic to combine different types of data sets and reason about them in a biologically meaningful way, by formalising in a rule-based representation the implicit reasoning of biologists, when considering such data sets. This largely qualitative method is very useful for combining different, large data sets, testing their consistency with known phenotypes, predicting phenotypes according to the pathway model and generating hypotheses that could be investigated further by more detailed experimental and quantitative computational approaches. Moreover, the ASP framework and the available solvers generate all possible solutions (answer sets) to a problem, given the input data at hand. Specific signals involving smaller segments of the pathway, such as the feedback mediated by ATG1, S6K and CHICO in the *Lnk* mutant, can be investigated by further experiments. For example, western-blot analysis can be performed in time-series experiments after pathway induction by ligand stimulation and their impact on longevity can then be studied by dynamic simulations. However, such experiments, validating our hypotheses, would benefit from the use of different biological systems, such as cell line models, instead of *in vivo* whole-organism models that have been used for the gene expression and lifespan assays.

In the majority of cases, primary effects reflect the impact of the perturbation on lifespan, whereas secondary effects seem to be parts of feedback mechanisms that lead to homeostasis, attempting to “correct” the effect of the experimental perturbation. When comparing the long-lived mutants against the *foxo* knock out, the only short-lived mutant, we generally observe opposite effects on differential expression of the insulin pathway components. For example, in all long-lived mutants we observe at least one *Ilp* per experiment being up-regulated, in order to stimulate the pathway and lead to FOXO inactivation by facilitating its retention in the nucleus. Conversely, in the short-lived experiment we observe down-regulation of *Ilps*. Similarly, the inhibitor of ILP2 and ILP5, IMPL2, is down-regulated in the *Lnk* mutant and up-regulated in the *foxo* mutant.

Moreover, studying the predicted signals in the different mutants associated with differences in longevity, we are able to generate hypotheses on how lifespan could be modulated within the pathway framework, not only per mutant, but also across mutants. Interesting questions for further investigations are (1) whether the primary and secondary effects, as presented here, represent opposing contributions to the overall longevity phenotype we observe in the survival studies and (2) whether it would be possible to quantify these effects and identify a relationship between each signal and difference in lifespan. Moreover, our current pathway model ignores the contribution of other pathways and processes, e.g. growth/autophagy, to lifespan by solely examining lifespan as a result of FOXO activation. It could be possible, in the future, to integrate methods that employ dynamic simulations, such as the one used by Dalle Pezze *et al* [51], in order to delineate the complex effects of the different signals on FOXO for each mutant.

Our application is limited by the lack of kinetic information on the pathways and also by the experimental data sets, which contain mRNA levels, making the quantitative analyses of signalling pathways impossible. Moreover, we integrate pathway connections derived by different experimental methodologies, while the accuracies of these connections are ignored by the analyses, because they are sometimes unavailable or unsuitable for simulations. However, since we have been strict in only selecting connections that have been experimentally verified, our method gains in strength by the use of larger but qualitative pathway diagrams thus making their analysis possible in a qualitative fashion, as opposed to the computational difficulties faced by quantitative methods and discrete logic-based methods, to simulate larger pathways.

It would be an interesting exercise to test on a smaller example the performance of tools implementing Markov Logic Networks, e.g. Alchemy by Domingos *et al* [52], which combine statistical relational learning with knowledge-based model construction. We believe that the improvement of the formal logic-based reasoning methods and their integration with statistical methods will lead to the development of hybrid approaches that can leverage quantitative information where available, but also take advantage of the rich representational language to combine disparate and qualitative data sets in order to generate more accurate and meaningful biological hypotheses.

The rule-based model itself is general and can be used to reason about different types of pathways, other than the IIS and TOR pathway and relate these to phenotypes other than longevity. In the future, we will enable the import of user-defined pathways to the web-service, so that users could relate their molecular data sets to their pathways of interest. It is important that for the inclusion of different types of pathway models we develop further the web-service, so that it is compatible with standard pathway formats, such as BioPAX and SBML.

Our current pathway model aims to represent the topology of the interactions without including constraints on the conditions or tissues, where these interactions take place. Such information, once available in sufficient detail to have an impact on the analyses, could potentially be collected and included in the rule-based logic program. We anticipate that the modular nature of our model will enable it to expand and combine pathway processes at different levels of detail (e.g. undirected Protein-Protein Interaction Networks), as new information on tissues, granularity of pathway interactions becomes available. Currently, the longevity phenotype is considered in a discrete fashion as “long-lived”, “unchanged”, or “short-lived”. In the future, we aim to integrate the methods implemented in the web-service SurvCurv (manuscript in preparation) with the methods in NetEffects in order to achieve a more quantitative and full representation of longevity phenotypes.

As it stands, the pathway model and the ASP methods can be used to investigate the signalling effects on the IIS pathway by longevity experiments that involve exposure to environmental stress conditions or perturbations of other genes, not included in the IIS pathway. A requirement is that some of the components in the pathway model must be differentially expressed in these experiments, therefore implicating the IIS pathway in the response to the experimental condition. For example, these could include dietary restriction experiments or perturbation of other transcription factors that have also been shown to alter ageing. In the future we also aim to generalise the web-service to include the longevity-related pathways of other model organisms used in ageing research, such as *C.elegans* and *M.musculus*, with the aim of performing cross-species data analyses to investigate how the predicted signals in equivalent mutants differ across species.

## Supporting Information

Supplementary File S1 **All paths inferred per experiment.**
(XLS)

Supplementary File S2 **The developed logical theory (code).**
(TXT)

Supplementary File S3 **The longest common sub-paths per experiment.**
(XLS)

Supplementary File S4 **Analysed microarray data sets (LIMMA output).**
(XLS)

Supplementary File S5 **Supplementary Methods.** Description of Answer Set Programming and the logical theory we developed.
(PDF)
