# Supplementary material for: Using Answer Set Programming to Integrate RNA Expression with Signalling Pathway Information to Infer How Mutations Affect Ageing
Source: PLoS One. 2012 Dec 10;7(12):e50881. doi: 10.1371/journal.pone.0050881 (PMC3519537; doi:10.1371/journal.pone.0050881)
Supplement: Supplementary File S5 — Supplementary Methods. Description of Answer Set Programming and the logical theory we developed. (PDF) [file pone.0050881.s005.pdf]

Supplementary Methods

Answer Set Programming

Answer Set Programming (ASP) is a computational method for solving difficult combinatorial problems. It is a type of declarative programming, meaning that it expresses `what' a program should do, by describing the logic of the computation, in contrast to describing `how' a program should do a task. ASP is based on the stable model (answer set) semantics of logic programming. A typical ASP system uses a program that computes stable models (answer sets), called an `Answer Set Solver'. Given a knowledge base of ground terms and a set of rules (logic program) relating general concepts that include the types of information in the knowledge base, an ASP solver will find minimal answer sets (stable models) that satisfy the rules in the logic program and consist of a subset of the ground terms in the knowledge-base, together with new ground terms that can be inferred by evaluating the rules with the available ground terms. This process is usually performed in two steps. First, a *grounder*, a program that substitutes all the variables of the rules in the logic program with the available values (ground terms) in the knowledge base, is used in a process referred to as `grounding'. Second, the ASP *solver* accepts the ground program produced by the grounder and computes the answer sets by checking their consistencies with the logic program and the integrity constraints. The answer sets are the stable models of the original program and represent the minimum number of ground terms that could satisfy the rules and integrity constraints.

We use the implementations of Gebser *et al* [1], with `gringo', [2], as a grounder and `clasp', [3], as a solver in the two-step process of `grounding' and `solving'. The above references, in addition to implementation details, also include a more extensive description and definitions of Answer Sets and Answer Set Programming.

The knowledge base

In this application, the knowledge base consists of information derived from the pathway described in the Results Section and the gene expression data for the experiment to be evaluated. The description of the pathway model in GraphML is converted into predicates, the relations between the different entities that can be read by the ASP programs. Similarly, information about the experiment, such as type of mutation, differentially expressed genes and mutated genes are also converted into predicates, as shown below. Information on the experiment is retrieved from files containing pre-analysed microarray data sets and information on the experimental perturbation is submitted by the user.

First, the information of the pathway model is expressed as relations between entities, such as proteins, complexes, or a complex and a protein. Here, the entities are represented by `a' and `b', where `a' acts on `b'. These entities are expressed as lower-case constants, consisting of the protein and protein complex names that are present on the pathway map. Upper case entities show variables

that can be substituted by values of the different constants, during the process of grounding:

```
inhibits(a,b)
induces(a,b)
```

Moreover, if a protein `a' is in a complex `c', then we express this relationship as:

```
in_complex(a,c)
```

`Cofactor' relationships, where a specific protein needs to be present for an interaction of two different proteins to take place is expressed as:

```
false :- inhibits(a,b), inactivates(X, c)
```

meaning that protein `a' cannot inhibit `b' if `c' is inactivated in the experiment. Inactivation can happen by interaction with another protein `X' (as inferred in the current experimental conditions) or it is assumed to happen if the mRNA of `c' is down-regulated in the expression experiments.

The knowledge base also contains a description of the experimental perturbation and results that lead to an up-regulation or down-regulation of a gene product `a' in experimental conditions `e':

```
upregulates(e,a)
downregulates(e,a)
```

Rules of gene interactions

The rule-based theory (ASP program) consists of a set of rules, of the form *h:-b*, meaning that if *b* is true, then *h* is true. *b* is known as the body of the rule and can consist of one or more comma-separated predicates, meaning that all these predicates must be true for *h* (the head of the rule) to be true. The program describes how a gene mutation or a difference in gene expression could affect the function of the corresponding signalling component (signalling protein or complex). We assume that if a gene is `down' (i.e. knocked out by perturbation or down-regulated as a result of a perturbation), then the functionality of its end-product will be impaired and will result in reduction of the effect on components it directly inhibits or induces. Components that under different conditions get activated will now be `inactivated' and components that normally get inhibited will now be `activated'. These relationships are expressed using the following rules:

```
activates(G,X) :- inhibits(G,X), downregulates(E,G).
activates(G,X) :- induces(G,X), upregulated(E,G).
```

In the first rule, at the *body* it is stated that if a gene `G' is down-regulated in an experimental condition `E' (according to the experimental results) and its protein product (`G') inhibits a protein `X' (according to the pathway model), then (*head* of the rule) `X' will be activated (by `G'). The second rule shows how a

gene `X' can be activated in a different way, by being induced by an up-regulated gene. There are similar rules defining the effects on inactivation of proteins by genes that are up- or down-regulated (see supplementary files), completing the four different ways under which a protein can be influenced by gene expression. These four rules comprise the base-cases of more general recursive versions that facilitate the inference of the effects on signalling between proteins across the entirety of the pathway in the knowledge base.

An example of the recursive cases of the rules is shown below:

```
activates(G,X) :- inhibits(G,X), inactivates(Y,G).
activates(G,X) :- induces(G,X), activates(Y,G).
```

The first rule states that if a protein `X' is inhibited by a protein `G' and `G' itself is inactivated (by a protein `Y'), then `X' will be activated (by `G'). Another way that a protein `X' can be activated is via activation of a protein `G', which then induces `X'. The rest of the cases of these rules are available from the complete theory (logic program), available in supplementary files.

Finally, we express the effects of complexes on signalling with rules stating that if one or more of their components are down-regulated, then the whole complex is down-regulated and cannot be activated by any of the above rules. In these cases we make the assumption that the complex cannot form because one or more of its components is down-regulated and therefore, subsequent signals, downstream of that complex are impaired.

```
inactivated_complex(E,X):-in_complex(G,X), downregulates(E,G).
```

where `E' is the experiment, `G' is a component of complex `X'.

Second Step: Path Finding

Our ASP analysis outputs a set of ground terms of the form `activates(a,b)' or `inactivates(a,b)'. These ground terms represent the inferred effect of `a' on `b' at the protein signalling level. The rules in the ASP theory have been defined in such a way that a component `a' activates or inactivates another component `b', provided that `a' or `b' are not differentially expressed in a manner that would contradict their effect on signalling. For example, we cannot infer that `a' activates `b' if `a' or `b' or both are downregulated at the gene expression level. This enables us to identify all the possible signals present in the data set at hand. However, it requires a second step of `path sorting', where we identify every route, `path', that starts from a component that is perturbed or differentially expressed in the experiment and leads to changes in lifespan as a result of FOXO regulation. We define paths as:

relation(C, X), relation(X,Y) ... relation(Z, Longevity).

where C is the altered (differentially expressed or perturbed) component, `relation' stands for either `activates' or `inactivates' and X,Y,Z are other proteins or complexes.

The path-searching algorithm takes as input the set of answers of the ASP step and the description of the pathway in logical notation (knowledge base). It proceeds in a stepwise manner through the list of the perturbed and differentially expressed components, and for each one of those identifies a set of continuous paths from the perturbed/ differentially expressed component to longevity.

References

1. Gebser M, Kaminski R, Kaufmann B, Ostrowski M, Schaub T, et al. (2011) Potassco: The Potsdam Answer Set Solving Collection. AI Communications 24: 105–124.

2. Gebser M, Kaminski R, K¨onig A, Schaub T (2011) Advances in gringo series 3. Logic Programming and Nonmonotonic Reasoning .

3. Gebser M, Kaufmann B, Neumann A, Schaub T (2007) Conflict-driven answer set solving. In: IJCAI 2007, Proceedings of the 20th International Joint Conference on Artificial Intelligence.
